# Supplementary material for: Using Social Networks to Estimate the Number of COVID-19 Cases: The Incident (Hidden COVID-19 Cases Network Estimation) Study Protocol
Source: Int J Environ Res Public Health. 2021 May 26;18(11):5713. doi: 10.3390/ijerph18115713 (PMC8198250; doi:10.3390/ijerph18115713)
Supplement: Supplementary file 1 [file ijerph-18-05713-s001.zip › ijerph-1153833-supplementary.pdf]

Supplementary

Questionnaire

INCIDENT

Descrizione dello studio

Capire quante persone sono potenzialmente positive al COVID-19 è di fondamentale importanza in un momento di emergenza come questo per definire le iniziative di contenimento dell'epidemia.

L'Università degli Studi di Padova insieme all'Università degli Studi di Torino e la Prochild Onlus hanno ideato lo studio INCIDENT (hIddeN CovID-19 casEs Network esTimation), con il supporto tecnico gratuito offerto da Zeta Research.

INCIDENT si propone pertanto di quantificare il numero di persone con infezioni da COVID-19 non documentate da tampone positivo attraverso il presente questionario volontario, anonimo e gratuito.

Il questionario è composto di 10 domande, di cui 4 socio-demografiche, 6 riguardanti il COVID-19 e l'attuale situazione di emergenza, e l'ultima è una domanda casuale per stimare la dimensione della rete di conoscenze.

I dati raccolti saranno trattati ad esclusivi fini di ricerca e divulgazione scientifica.

Definizione di conoscenza

In questo studio per conoscenza intendiamo qualcuno con cui hai reciproca conoscenza, di vista o di nome, oppure qualcuno con cui hai avuto un contatto (di persona, via telefono o per corrispondenza) negli ultimi due anni e che possa ripetersi oggi[1,2].

DOMANDE

1) Quanti anni hai?
2) Sesso
3) Paese d'origine
4) In che provincia risiedi attualmente?

Domande sulla sintomatologia

5) Quante persone conosci che hanno avuto almeno un sintomo come febbre (>37.5°C), tosse secca, mal di gola, congestione nasale, malessere, mal di testa, dolore muscolare nelle ultime due settimane?

Domande sull'esposizione

6) Quante persone conosci che sono risultate positive al tampone per il COVID-19?

Domande sulle misure di sicurezza

7) Quante persone conosci che condividono l'abitazione con soggetti in isolamento a causa di manifestazione di sintomi simil-influenzali?

Domande relative agli spostamenti

8) Quante persone conosci che si sono spostate da una regione all'altra per rimanervi stabilmente dopo l'emanazione del decreto #iorestoacasa (D.P.C.M. 08.03.2020)?

Domanda random per definire il social network

9) Domanda per social network (random)

REFERENCES

1. Bernard HR, Hallett T, Iovita A, et al. Counting hard-to-count populations: the network scale-up method for public health. *Sex Transm Infect*. 2010;86(Suppl_2):ii11-ii15. doi:10.1136/sti.2010.044446

2. Bernard HR, Johnsen EC, Killworth PD, Robinson S. Estimating the size of an average personal network and of an event subpopulation: Some empirical results. *Soc Sci Res*. 1991;20(2):109-121.
